# Supplementary material for: Histone methyltransferase SUV39H1 regulates the Golgi complex via the nuclear envelope-spanning LINC complex
Source: PLoS One. 2023 Jul 12;18(7):e0283490. doi: 10.1371/journal.pone.0283490 (PMC10337926; doi:10.1371/journal.pone.0283490)
Supplement: S1 File — (PDF) [file pone.0283490.s001.pdf]

Nishino et al., Supplementary Figure 1
SUV39H1 associates with the morphology of the Gogli complex

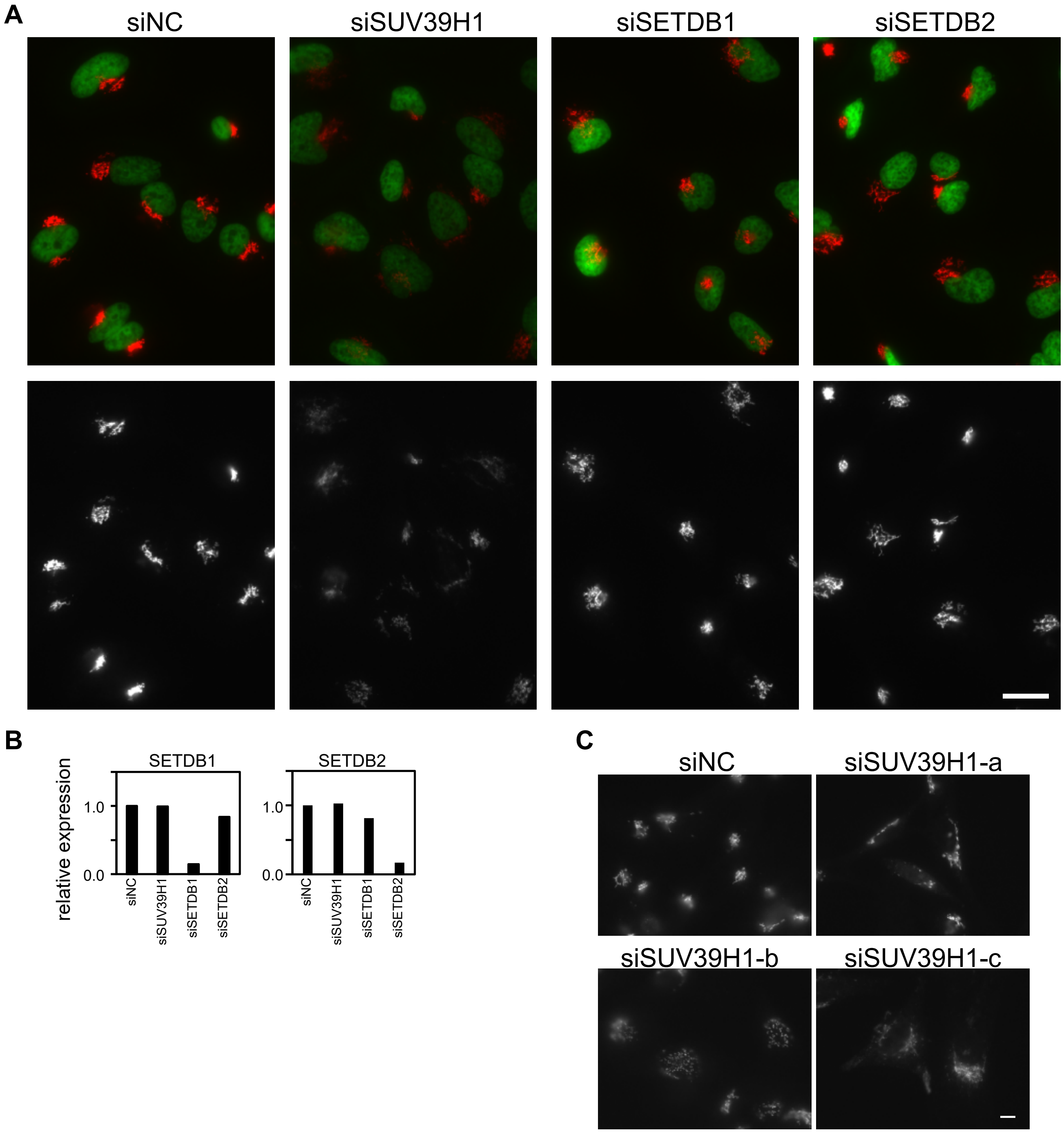

Nishino et al., Supplementary Figure 2

Mitotic Golgi dispersion and SUV39H1 depletion-induced Golgi dispersion are mediated by different mechanisms

A

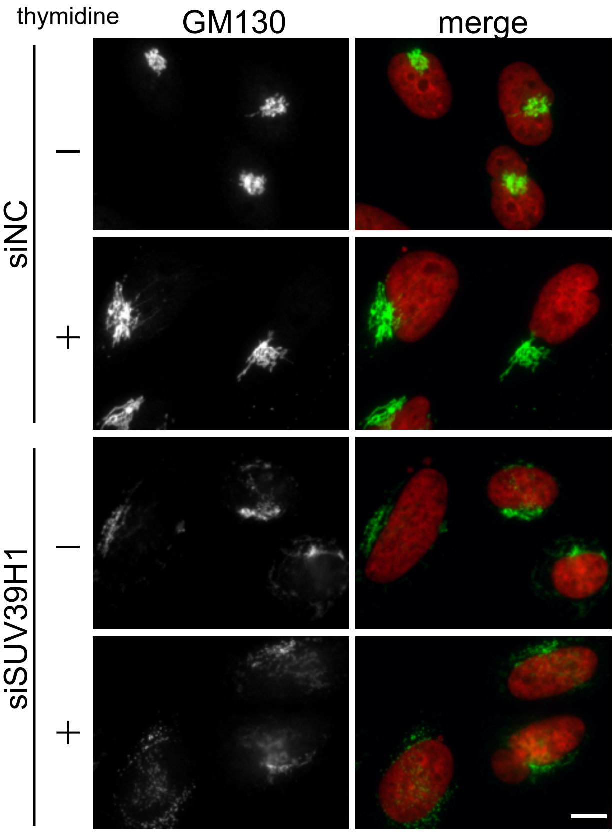

B

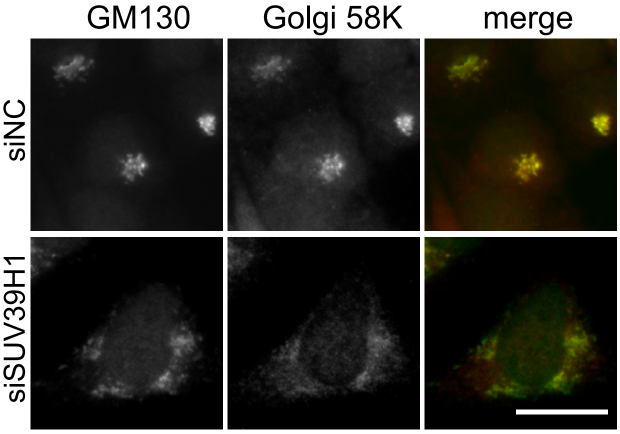

Nishino et al., Supplementary Figure 3

SUV39H1 depletion impairs Golgi complex integrity but does not affect MT or membrane trafficking

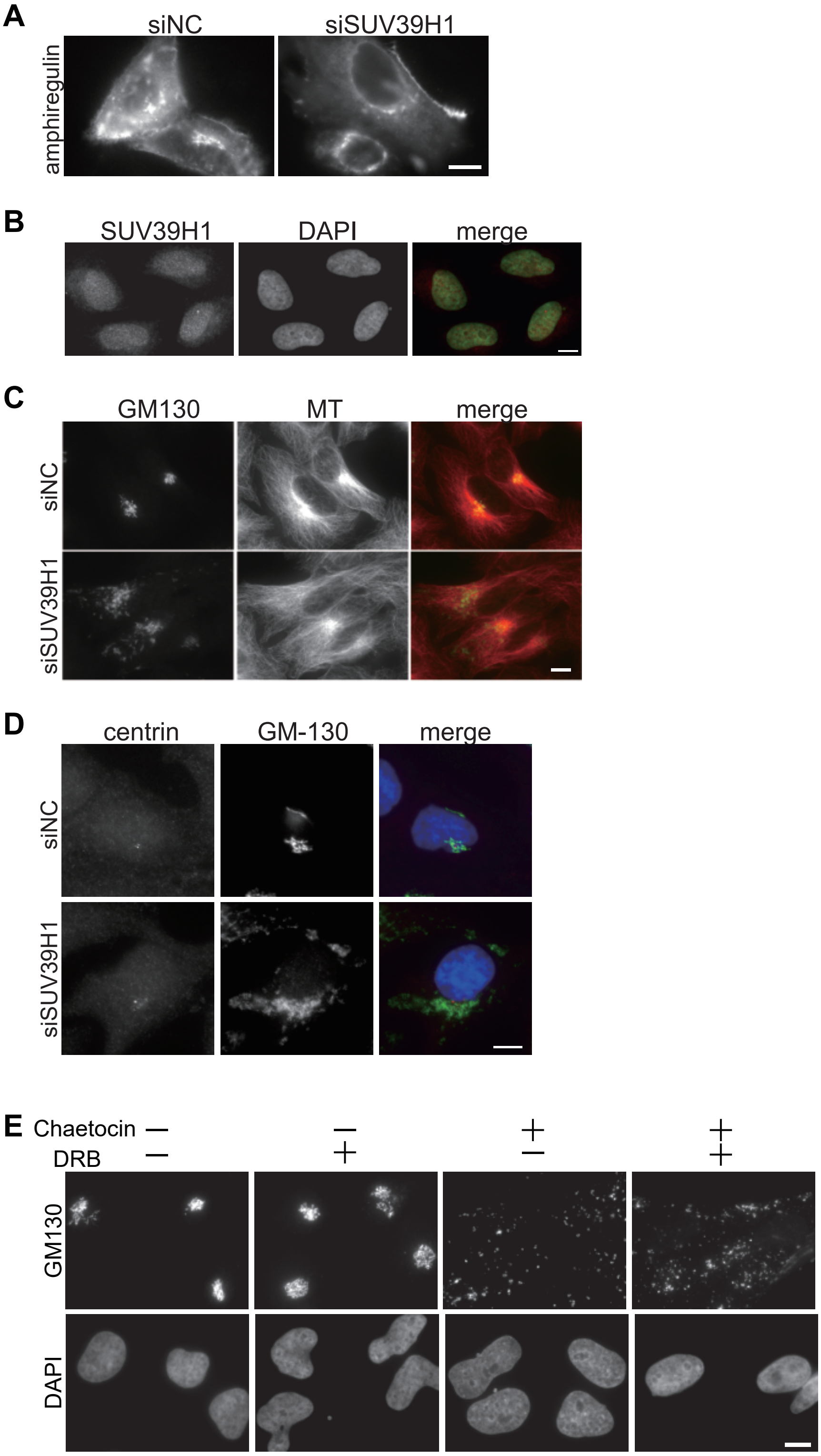

NIshino et al., Supplementary Figure 4

DN-KASH does not affect the perinuclear accumulated Golgi complex

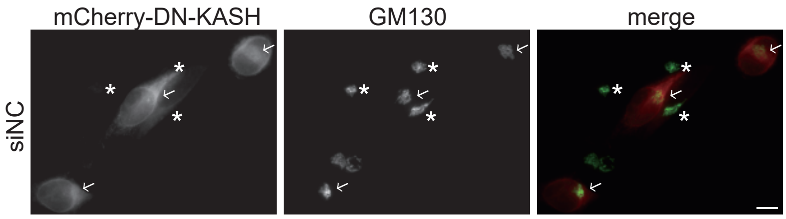

NIshino et al., Supplementary Figure 5

SUN1 protein expression in the SUV39H1-depleted cells

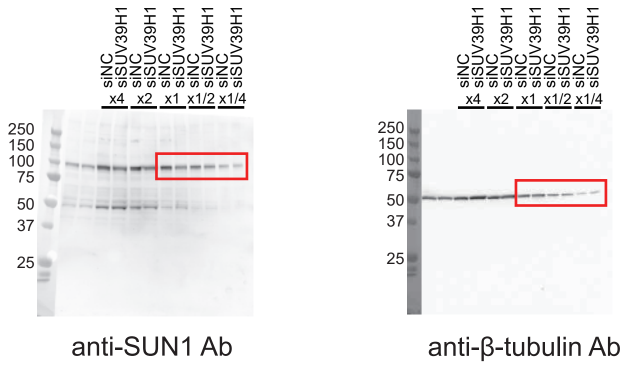

NIshino et al., Supplementary Figure6

SUN2 interacts with H3K9me3

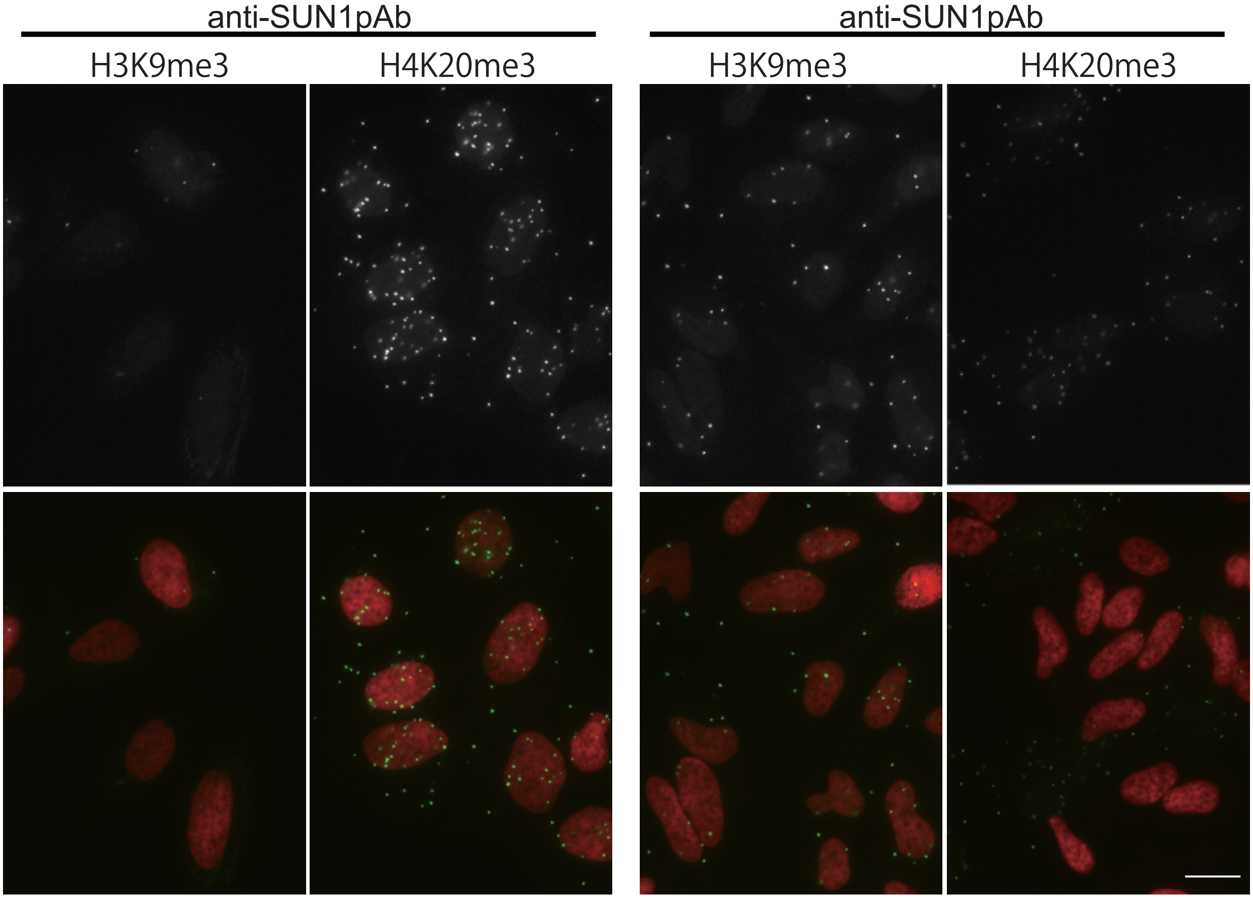

Supplemental Table 1. Genes regulated by SUV39H1 in HeLa cells

Genes upregulated in HeLa cells lacking SUV39H1

Name	RefSeq_ID	Description	Average ratio
HMOX1	NM_002133	Heme oxygenase 1	9.80
GDF15	NM_004864	Growth/differentiation factor 15 Precursor	8.00
PTGS2	NM_000963	Prostaglandin G/H synthase 2 Precursor	6.69
NUPR1	NM_001042483.1; NM_012385.2	Nuclear protein 1	5.81
GFPT1	NM_002056.2	Glucosamine--fructose-6-phosphate aminotransferase	4.54
SLC16A6	NM_004694.3	Monocarboxylate transporter 7	4.10
C1orf43	NM_001098616	Uncharacterized protein	3.27
CXCR4	NM_001008540	C-X-C chemokine receptor type 4	3.07
DNAJB6	NM_058246	DnaJ homolog subfamily B member 6	2.97
SGK1	NM_005627	Serine/threonine-protein kinase Sgk1	2.91
PFN2	NM_053024	Profilin-2	2.71
HSPB8	NM_014365	Heat shock protein beta-8	2.70
MARVELD1	-	MARVEL domain-containing protein 1	2.68
C11orf75	NM_020179	UPF0443 protein C11orf75	2.67
NUTF2	NM_005796	Nuclear transport factor 2	2.67
CREM	NM_182721	cAMP-responsive element modulator	2.63
IER3	NM_003897	immediate early response 3	2.61
MGMT	NM_002412	Methylated-DNA--protein-cysteine methyltransferase	2.51

Genes downregulated in HeLa cells lacking SUV39H1

Name	RefSeq_ID	Description	Average ratio
RRM2	NM_001034; NM_001034	Ribonucleoside-diphosphate reductase subunit M2	0.19
C17orf89	NM_001086521.1	Uncharacterized protein	0.21
FLII	NM_002018.2	Protein flightless-1 homolog	0.22
RFXANK	NM_134440	Protein flightless-1 homolog	0.23
sBRP44	NM_015415	DNA-binding protein RFXANK	0.24
RPE	NM_199229	Brain protein 44	0.26
ARL6IP5	NM_006407	Ribulose-phosphate 3-epimerase	0.27
TAF9B	NM_015975.4	PRA1 family protein 3	0.28
WDR1	NM_005112.4	Transcription initiation factor TFIID subunit 9B	0.28
TMEM123	NM_052932	WD repeat-containing protein 1	0.30
TFDP1	NM_007111	Porimin Precursor	0.31
RTN4	NM_153828; NM_207520	Transcription factor Dp-1	0.31
OSTM1	NM_014028	Reticulon-4	0.31
FBXO21	NM_033624	Osteopetrosis-associated transmembrane protein 1 Precursor	0.31
TNNT2	NM_000364; NM_001001431	F-box only protein 21	0.32
CAV1	NM_001753.3	Troponin T, cardiac muscle	0.32
TM2D3	NM_078474	Caveolin-1	0.33

Genes listed were up- or down-regulated more than 3 fold repeatedly (n = 3) in SUV39H1-knockdown cells (*, P < 0.05: **, P < 0.01).

.

Supplemental Table S1.

A. siRNA sequences (all siRNAs had 3’ overhangs)

Target regions	siRNA sense
SETDB1	CAAAGAUGGUGACCUGAUA<br>AAGAUGGGCUUUCAUGUUA<br>GCAAGGAGCUACUCUGUUG<br>GUAGAAAGCUCCCACAGUU
SETDB2	GAUCAGCCCUUUCAUCUCA<br>GCGUAUGCAUUGCUGAGUU<br>GAACUUCUCGGUGGUGGUA<br>CAGGAGGCCAUGAGCGCAU

B. List of primer sets used in this study

Target regions	
SUN1	5’-AAGTCAGAGAAATGGTGAAACTCC-3’<br>5’-TCACAAACTGTGATGAGAACCTCT-3’
SUV39H1	5’-GTCATGGAGTACGTGGGAGAG-3’<br>5’-CCTGACGGTCGTAGATCTGG-3’
SETDB1	5’-GCTCCGTGAAGCTATGGCTGCC-3’<br>5’-GGTAATCATAGGCAATATGGTT-3’
SETDB2	5’-TAAAACCCCTTGTGGAAGGAGT-3’<br>5’-AAGGCTGCATTCATAAATGCC-3’
